# In vitro physiological and antibacterial characterization of ZnO nanoparticle composites in simulated porcine gastric and enteric fluids

**DOI:** 10.1186/s12917-017-1101-9

**Published:** 2017-06-17

**Authors:** Marina S.R. Barreto, Cristina T. Andrade, Luiz Cláudio R. P. da Silva, Lúcio M. Cabral, Vânia M. Flosi Paschoalin, Eduardo M. Del Aguila

**Affiliations:** 1 Instituto de Macromoléculas Professora Eloisa Mano, Universidade Federal do Rio de Janeiro, Centro de Tecnologia, Bloco J, Rio de Janeiro, RJ 21941-598 Brazil; 2 Universidade Federal do Rio de Janeiro, Faculdade de Farmácia, Av. Carlos Chagas Filho, 373, CCS, Bloco L, sala 24, Rio de Janeiro, RJ 21941-902 Brazil; 3 Instituto de Química, Universidade Federal do Rio de Janeiro, Av. Athos da Silveira, Ramos 149, Bloco A, sala 545, Rio de Janeiro, 21941-909 Brazil

**Keywords:** Chitosan/alginate coatings, ZnO nanoparticles, Biological properties, Piglets

## Abstract

**Background:**

Diarrhea in piglets is one of the main causes of animal death after weaning; zinc oxide (ZnO) has been used in high doses for the control of this sickness. The aim of this study was to determine the physicochemical properties of ZnO nanoparticles synthesized and immobilized on a chitosan/alginate (CH/SA) complex and investigate the antimicrobial activity and in vitro release profile of zinc (Zn^2+^) from these new compounds. The ZnO nanoparticles composites were prepared and combined with CH/SA or CH/SA and sodium tripolyphosphate (TPP). The structure and morphology of the composites were analyzed by characterization methods such as X-ray diffraction, FTIR spectroscopy, thermogravimetric analysis, atomic absorption spectrophotometry and scanning electron microscopy.

**Results:**

The crystallite size of ZnO nano was 17 nm and the novel ZnO composites were effective in protecting ZnO in simulated gastric fluid, where Zn^2+^ reached a concentration six-fold higher than the levels obtained with the unprotected commercial-zinc oxide. In addition, the novel composites suggest effective antimicrobial activity against *Escherichia coli* and *Staphylococcus aureus.*

**Conclusions:**

The results described herein suggest that the novel nano composites may work as an alternative product for pig feeding as verified by the in vitro assays, and may also contribute to lower the zinc released in the environment by fecal excretion in animals waste.

## Background

For decades, farms have used pharmacological concentrations of zinc oxide (ZnO) to combat post-weaning diarrhea [1, 2]. Different modes of action for the antibacterial activity of ZnO have been proposed, one of them being the partial dissolution of the ZnO particles in the enteric region and the release of Zn^2+^ [3]. When ZnO is formulated to reduce this undesirable dissociation in the stomach, less ZnO may be added to the diet [4]. This is important, since much of the ZnO given to the piglets is excreted with their waste, causing accumulation of this metal in the environment [5, 6]. The incorporation of ZnO nanoparticles in polymer matrices can facilitate their gradual and sustained release [7].

Chitosan (CH) and alginate (SA) are two of the most commonly studied biopolymers, particularly for drug delivery [8]. CH is the partially deacetylated derivative of chitin, composed of repeating units of β-(1,4)-2-amino-2-deoxy-D-glucose and β-(1,4)-2-acetamido-2-deoxy-D-glucose [9]. SA is extracted from brown seaweeds and contains carboxylic acid groups [8], presents linear chains with blocks of 1–4–linked α-L-gluronic (G-block), β-D-mannuronic acid (M-block) residues, and blocks of alternated G and M residues [10].

In the present study, CH and SA, with and without the addition of sodium tripolyphosphate, were combined to encapsulate nanostructured ZnO particles aiming for the control of their enteric release. To protect ZnO from the gastric acid environment, the composite structures were designed to maintain acid-soluble chitosan in the internal phase of the complex. The physical, chemical and rheological characteristics of the resulting products were analyzed by infrared spectroscopy, X-ray diffraction, atomic absorption spectrophotometry, thermogravimetry and scanning electron microscopy. The antibacterial properties of the composites were tested against *Escherichia coli* and *Staphylococcus aureus* and the MICs were calculated. The in vitro release profile of Zn^2+^ from the composites in both gastric and enteric simulated fluids was also investigated.

## Methods

### Reagents and solvents

The commercial zinc oxide (ZnO) sample (D_XRD_ = 0.22 μm) was supplied by Brasóxidos Indústria Química Ltda. (Sertãozinho-Mauá, SP, Brazil). Chitosan (CH) (75%–85% DA), sodium alginate (SA), sodium tripolyphosphate (TPP) (85% PA) and zinc acetate dehydrate (99% PA) were purchased from Sigma-Aldrich Co. (St. Louis, MO, USA). Acetic acid (99.7% PA), sodium hydroxide (99% PA), hydrochloric acid (37% PA), hydrated potassium phosphate (P.A.) and sodium chloride were purchased from Vetec Química Ltd. (Rio de Janeiro, RJ, Brazil).

### Preparation of biopolymer solutions, zinc oxide dispersion and microcapsules

The CH solution at 2% (m/v) concentration was prepared by adding 2 g CH slowly to 100 mL of acetic acid 1% (*v*/v), under magnetic stirring. This solution was heated for 30 min and maintained for 12 h under stirring at room temperature. The SA aqueous solution at 2% (m/v) was prepared using 2 g of SA in 100 mL water with magnetic stirring for 12 h. The TPP solution at 3% (m/v) was prepared in 100 mL ultrapure Milli-Q water (18.2 MΩ·cm at 25 °C).

A zinc acetate dihydrate 0.2 M solution (13.2 g in 500 mL water) was prepared with Milli-Q water under magnetic stirring and at room temperature in a two-necked 1000 mL flask adapted to a heating mantle and a reflux condenser. The reaction mixture was refluxed for 30 min. After cooling, a 5 M NaOH solution was added dropwise, until achieving pH 11 [11], after which refluxing was continued for 3 h. The reaction product was decanted and the supernatant solution discarded. After successive washings with Milli-Q water, the product was oven-dried at 100 °C for 24 h, characterized and used for the preparation of the microcapsules.

Commercial ZnO (250 mg) was dispersed in water (Millipore, model Direct-Q3, São Paulo, SP, Brazil) at 4 g/100 mL composition, at 10.000 rpm with a Ultra-Turrax dispersing IKA T25 equipment (IKA®, São Paulo, Brazil) for 40 min, oven-dried at 100 °C for 24 h and characterized.

ZnO nanoparticles were added to 20 mL of acetic acid 1% solution in a 50 mL beaker, and manually dispersed until no more granules were observed. Subsequently, this dispersion was homogenized in a Turrax at 10,000 rpm for 30 min and 15 mL of the CH 2% solution was added, dropwise, with the aid of a Pasteur pipette, at a Turrax stirring speed of 5000 rpm. Then, 30 mL of the SA 2% solution was added, dropwise, still under stirring at 5000 rpm, resulting in ZnO nanoparticles and both CH and SA (ZnO/CA) and ZnO microparticles with CH, SA and TPP (ZnO/CAT), in which 15 mL of a TPP solution 3% in Milli-Q water were also added to the reaction mixture. The resulting mixtures were subjected to ultrasonic radiation for 15 min (40% amplitude, 1 s/1 s intervals, in ice baths) (750 W Sonics & Materials Inc., Newtown, CT, USA) and dried at 60 °C for further characterization.

### Physicochemical and morphological characterizations

The XRD curve for the samples was obtained on a Miniflex diffractometer (Rigaku Corporation, Osaka, Japan) operating at a CuK_α_ wavelength of 1.542 Å. The samples were exposed to the X-ray beam with the X-ray generator running at 30 kV and 15 mA. Scattered radiation was detected at ambient temperature in the angular region (2θ) of 2–80° at a rate of 3°/min and a step size of 0.05°. The diffractogram was smoothed (Savitsky-Golay, polynome = 2, points = 7) and the baseline was corrected. Scherrer’s equation (Eq. (1)) was used to estimate the average size of the ZnO crystallites.

1 $$ {\mathrm{D}}_{\mathrm{XRD}}=\mathrm{K}\lambda /\upbeta \cos \uptheta $$

where D is the average crystallite diameter in Å, K = 0.9 is the shape factor, λ = 1.5418 Å is the wavelength of the CuK_α_ radiation and θ is the angle of Bragg diffraction. The β value was determined by Eq. (2)

2 $$ {\upbeta}^2={\left({\mathrm{B}}^2-{\mathrm{b}}^2\right)}^{1/2} $$

where B is the full width at half maximum (FWHM) of the (101) reflection of ZnO and b is the FWHM of the (101) reflection of the quartz standard, which was taken directly from the software provided with the equipment.

The ZnO, ZnO/CAT and ZnO/CA samples were characterized by FTIR spectroscopy on a Perkin Elmer spectrophotometer, Frontier model (Waltham, MA, USA) using KBr disks, with accumulation of 20 scans and 2 cm^−1^ resolution. The KBr used to prepare transparent disks was permanently maintained in an oven at 50 °C. Samples were thoroughly dried and carefully weighed (2 mg) before grinding with 100 mg of KBr.

Rheological measurements were performed at 25 °C using a controlled AR2000 stress rheometer (TA Instruments Inc., New Castle, DE, USA), fitted with a cone-and-plate geometry (2° cone angle, 40 mm diameter, 54 μm gap). The strain sweep was measured first as the evolution of the complex modulus at 6.28 rad s^−1^ for the determination of the linear viscoelastic region. Subsequently, the frequency sweep (mechanical spectra) was measured from 10^−1^ to 10^2^ rad s^−1^ (at a strain value of 1%), within the viscoelastic region.

Thermogravimetric analysis (TGA) was performed under a controlled N_2_ atmosphere using a Q500 thermoanalyzer equipment from TA Instruments (New Castle, DE, USA). The measurements were performed at a heating rate of 10 °C/min from room temperature to 700 °C.

Zinc contents were determined according to the standard AOAC method (2005) using a Varian AA280 atomic absorption spectrometer (Les Ulis, France). Each sample was heated at 550 °C and the ash boiled with 10 mL of 20% HCl in a beaker and then filtered into a 100 mL standard flask. All samples were analyzed in triplicate.

Scanning electron microscopy (SEM) was used to visualize the samples with a FEI Quanta™ 400 (Hillsboro, OR, USA) scanning electron microscope, at the acceleration voltage of 20 kV. The samples were vacuum-coated with gold before measurements.

The particle size distribution (PSD) of the samples was determined by laser diffraction, using the Masterisizer 2000 Malvern equipment (Malvern Instruments, Malvern, UK). The samples were dispersed in water on a Hydro 2000SM apparatus until the laser obscuration index reached 10–12%. The PSD values for the samples were determined in triplicate and were expressed as equivalent volume diameters at 10% (d_10%_), 50% (d_50%_) and 90% (d_90%_) of the cumulative volume, as the average of the diameter values (D_4, 3_) and Span. The Span values indicated the particle polydispersity, and were calculated according to Eq. (3).

3 $$ \mathrm{Span}={\mathrm{d}}_{90\%}-{\mathrm{d}}_{10\%}/{\mathrm{d}}_{50\%} $$

### Bioactivity of ZnO complexes

Two solutions were prepared: simulated gastric juice solution (SGS) with 2 g of NaCl in 7 mL of HCl, with pH adjusted to 2.5, and simulated enteric juice solution (SES) using 6.8 g of KH_2_PO_4_ in 250 mL distilled water, adding 77 mL of 0.2 N NaOH at pH 6.8 and distilled water to complete the volume of 1 L for each [12].

The assays were performed in triplicate, in 250 mL beaker, using 175 mL of SGS or SES with 10 mg of each composite. This was added to 25 mL of the same solution into previously hydrated dialysis membranes (MEMBRA-CEL® dialysis tubing, MWCO 7000, 34 mm, 14 × 100 CLR). The systems were kept at 39 °C under gently magnetic stirring and the solutions (SGS or SES) and 15 mL aliquots were collected in different time intervals (0, 15, 30 and 45 min) and the solutions were replenished with the same volume. Aliquots were stored and submitted to AAS analysis to determine the amount of Zn ^2+^ released each time in the simulated media.

The minimum bacteriostatic concentration (MBC) evaluation of commercial ZnO, ZnO nano, ZnO/CAT and ZnO/CA samples was performed by the well diffusion method and by liquid growth-inhibition assay against *Escherichia coli* DH5α (strain 00379 INCQS-Fiocruz) and *Staphylococcus aureus* ATCC 6538 (INCQS-Fiocruz), Gram negative and Gram positive bacteria*,* respectively. The same assay was performed to investigate the antimicrobial activity of CH used in the formulation of the samples. The strains were grown in LB medium pH 7.0 (Luria-Bertani BD™) in an orbital shaker at 200 rpm, at 37 °C, for 24 h. The cellular density was adjusted in a saline solution (0.8% of NaCl) where the turbidity equivalent to McFarland 0.5 standard (1.5 × 10^8^ CFU/mL) was used as an inoculum in the presence of the composites at increasing concentrations, at 37 °C for 18 h at 200 rpm. Subsequently, cells were serially diluted in saline solution (0.8% of NaCl), plated on solid LB, and incubated at 37 °C for 18 h. Colony-forming units were counted. The MBC was taken as the concentration at which 100% growth inhibition was observed.

## Results

**Fig. 1:**
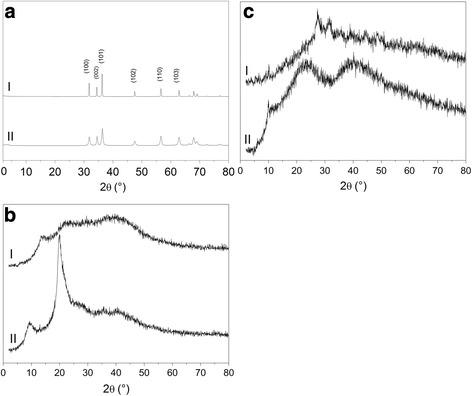
X-ray diffractogram in the 2θ region of 2.0° to 80° for the (**a**) I- Commercial ZnO, II- ZnO nano synthesized sample, (**b**) I- sodium alginate and II- chitosan, (**c**) I- ZnO/CAT and II- ZnO/CA

The crystallite size of nano and commercial ZnO (D_XRD_ = 17 nm and 0.22 μm, respectively) was calculated from Scherrer’s formula applied to the most intense reflection (101), as observed in the diffractogram displayed in Fig. 1a. It was observed that the reflections obtained for nano ZnO (trace II) were less crystalline with wider bases, characteristic of nanoparticles. Figure 1c shows that ZnO nanoparticles reduced the crystallinity when comparing to chitosan (CH) (Fig. 1b, trace II), as reflections were weaker and wide, as observed in a previous study [13]. Three strong reflections could be noticed, displayed in Fig. 1a between the angles of 2θ = 30° and 40°, corresponding to ZnO, but the diffraction corresponding to ZnO/CAT and ZnO/CA samples were very small, revealing the intense reduction in the orientation of crystallinity (Fig. 1c). It should also be noted that the ZnO/CAT samples encapsulated 10.2% of zinc and had a higher crystallinity than the ZnO/CA samples, with 12.6% Zn.

**Fig. 2:**
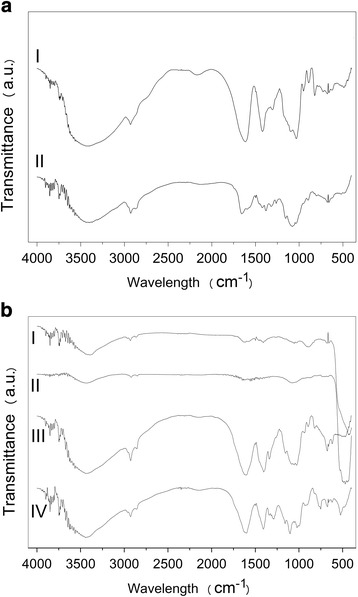
Infrared spectra obtained for (**a**) sodium alginate (trace I) and chitosan (trace II); (**b**) ZnO nano (trace I), commercial ZnO (trace II), ZnO/CAT (trace III) and ZnO/CA (trace IV)

The FTIR spectrum for SA (Fig. 2a, trace I) shows intense absorption bands with maximums at 3402, 2920, 1613, 1419 and 1032 cm^−1^. These bands were attributed to stretching vibrations of O-H, C-H bonds, asymmetrical and symmetrical stretching vibrations of the carboxylate groups and, finally, to C-O-C bonds from the polysaccharide structure. For the CH samples, the broad absorption with maximum at 3401 cm^−1^ was assigned to -OH and -NH_2_ stretching vibrations (Fig. 2a, trace II). The absorptions at 1657 cm^−1^ and 1594 cm^−1^were attributed, respectively, to the amide I (C = O stretching) and amide II (C–N stretching and C–N–H bending vibrations) from amide functional groups in the solid state. In this region (1650–1580 cm^−1^), the absorption attributed to the N-H bending (scissoring) vibration would be expected to appear. The strong absorption at 1094 cm^−1^ was attributed to the C-O-C bond from the polysaccharide structure.

Figure 2b displays the spectra for nano ZnO (trace I), the product obtained by encapsulating ZnO in the CH/SA complex (ZnO/CA, trace IV) and by encapsulating ZnO in the CH/SA complex reinforced with TPP (ZnO/CAT, trace III). For ZnO/CA (trace IV), the absorption at 471 cm^−1^, which also appeared in the ZnO nano (trace I), in commercial ZnO (trace II) and in ZnO/CAT spectra, were attributed to the Zn-O stretching vibration. This result pointed to the incorporation of ZnO to the CH/SA and CH/SA/TPP complexes.

**Fig. 3:**
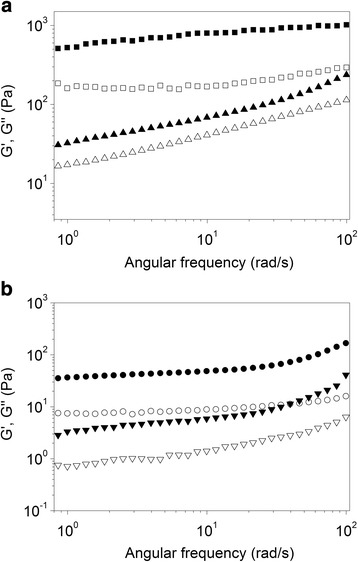
Variation of the storage modulus (G’, full symbols) and loss modulus (G”, open symbols) values as a function of frequency. **a** microparticles without ZnO, CAT (squares), CA (triangles); **b** microparticles with ZnO, ZnO/CAT (circles) and ZnO/CA (triangles)

The dynamic rheological results are displayed in Fig. 3 with the mechanical spectra for the SA/CH (CA) and SA/CH/TPP (CAT) complexes shown in Fig. 3a. The elastic behavior (G’ > G”), observed for both products, along the range of studied frequencies, revealed the formation of a tridimensional network.

**Fig. 4:**
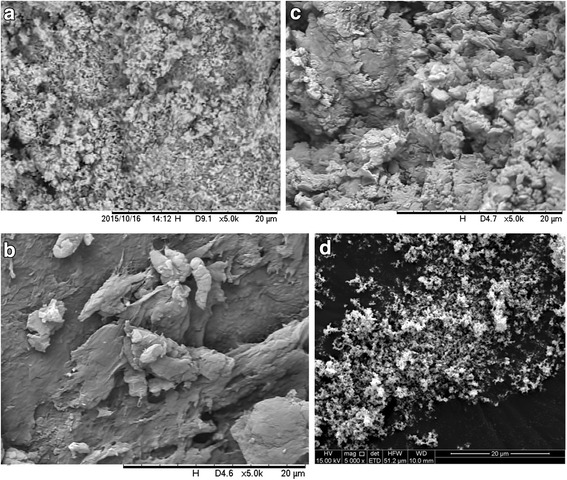
SEM micrographs of the morphology of microparticles. **a** ZnO nano, **b** ZnO with CH, SA and TPP sample (ZnO/CAT), **c** ZnO with CH and SA sample (ZnO/CA) and **d** commercial ZnO

Surface morphologies by SEM analysis of the nano and commercial ZnO, ZnO/CAT and ZnO/CA are displayed in Fig. 4. The surface morphology of pure ZnO nanoparticles (Fig. 4a) showed a fine powder with strong aggregation, characteristic of nanomaterials. In the ZnO/CAT micrograph, microparticles can also be observed as influencing the crosslinking of the biopolymer and the surface structure of the material (Fig. 4b).

**Fig. 5:**
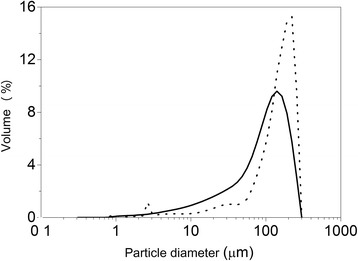
Size distribution for ZnO**/**CA (*dotted* line) and ZnO**/**CAT sample (*continuous* line) microparticles

The presence of 78% Zn^2+^ in the new pure synthesized ZnO nano and 99% in the commercial ZnO was observed, while the ZnO/CAT and ZnO/CA composites showed 10.2 and 12.6%, Zn^2+^, respectively by atomic absorption spectrophotometry (AAS). The particle size of each composite is displayed in Table 1 and Fig. 5. In all measurements, the ZnO/CAT samples were shown to be a smaller particle than the ZnO/CA samples. When the mean values were evaluated, a value of 114.32 μm of ZnO/CAT against 162.48 μm ZnO/CA was observed. However, the poly dispersion index expressed in Span value for this sample was higher (1.80 μm), revealing a less homogeneous distribution of particles in comparison with the ZnO/CA, that had a smaller span value (1.24 μm) (Fig. 5).

**Table 1:** Diameters of the ZnO**/**CAT and ZnO/CA microparticles

Sample	d_10%_ (μm)	d_50%_ (μm)	d_90%_ (μm)	d_4,3_ (μm)	Span (μm)
ZnO/CAT	18.59	109.63	216.46	114.32	1.80
ZnO/CA	47.92	169.11	257.16	162.48	1.24

**Fig. 6:**
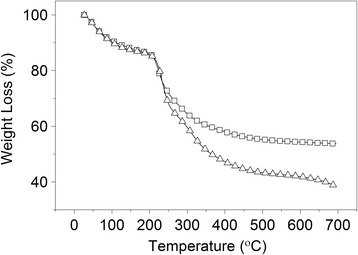
TGA curves for ZnO/CAT and ZnO/CA samples at a heating rate of 10 °C/min

**Fig. 7:**
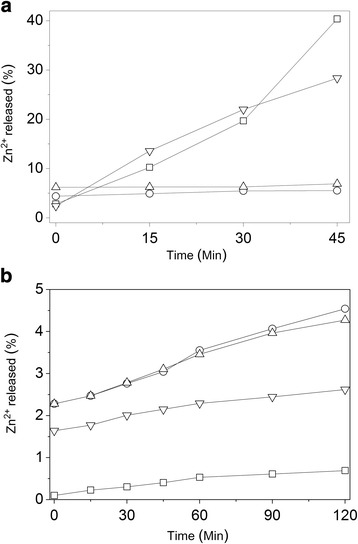
In vitro Zn^2+^ release profile from ZnO commercial, ZnO nano, ZnO/CAT and ZnO/CA in gastric (SGF) (**a**) and enteric (SEF) (**b**) simulated fluids  ZnO commercial,  ZnO,  ZnO/CAT,  ZnO/CA

The amount of inorganic residues (after heating to 700 °C) can be observed in the TGA curve, that indicated mineral matter content of 40%, approximately, for ZnO/CA, and 55% for ZnO/CAT (Fig. 6). The largest residue observed in the ZnO/CAT sample is due to phosphorus (P) from the TPP and Zn^2+^ present in ZnO. However, the results of the AAS Zn^2+^ quantification indicated that the ZnO/CAT samples have lower Zn^2+^ concentration (10.2%) compared to the ZnO/CA samples (12.6%). The in vitro release assay demonstrated that the ZnO samples complexed with CH, SA and TPP (ZnO/CAT) and no TPP (ZnO/CA) were effective in protecting Zn^2+^ in simulated gastric medium, as displayed in Fig. 7a. In SGF, at time zero, nano and commercial ZnO samples had the lowest release of Zn^2+^ between the 4 tested samples, however, the first 15 min revealed an increase of 11.2%, from 2.4 to 13.6% (commercial ZnO) and 7.4%, from 2.8 to 10.25% (ZnO nano). The release of ZnO/CA and release of the ZnO/CAT sample remained constant (4.5% and 6.2%) during the same period. From 15 min onwards, release by the ZnO nano and commercial sample increased and became more intense, reaching 40.4% and 27%, respectively, at 45 min. It was verified that despite the nano ZnO contain less Zn^2+^ in its formulation (78%) when compared to the commercial ZnO (99%), it released 1.5 times more at 45 min. This behavior was not observed in the protected samples, ZnO/CAT and ZnO/CA. Both samples had a low and constant release taxa as shown in Fig. 7a, 8.0 (5.3%) to 10.7 (6.9%) times lower than that observed in the ZnO nano sample. Figure 7b indicates that, at the beginning of the test using SES, nano ZnO released less Zn^2+^ (0.1%) than the ZnO/CAT (2.3%) and ZnO/CA (2.3%) samples and remained as such until the end time point (120 min) of the experiment, when a release of 4.2% and 4.5% was recorded, respectively. While commercial ZnO showed an initial release of 1.64% Zn^2+^ and a gradual increase of 2.62% tea at 120 min.

The antimicrobial activity of nano and commercial ZnO was tested. ZnO/CA sample exhibited the lowest MBC for both tested bacteria (*E. coli* and *S. aureus*) (Tables 2 and 3), where 1 mg/mL of ZnO/CA sample (126 μg/mL Zn^2+^ incorporated) provoked a total inhibition of *E. coli* and *S. aureus* growths. The ZnO/CAT sample inhibited *E. coli* growth at 2.25 mg/mL (229.5 μg/mL Zn^2+^ incorporated) and *S. aureus* at 2 mg/mL (204 μg/mL Zn^2+^ incorporated). When compared to the activities using the pure ZnO, *E. coli* growth was inhibited by 350 μg/mL (273 μg/mL Zn^2+^ incorporated) and *S. aureus* growth by 250 μg/mL (195 μg/mL Zn^2+^ incorporated), showing that the incorporated Zn^2+^ concentration is lower in the chitosan and alginate composites. On the other hand, the commercial ZnO showed the worst antimicrobial activity compared to other compounds, with a MBC of 500 μg/mL (495 μg/mL Zn^2+^ incorporated) and 650 μg/mL (643.5 μg/mL Zn^2+^ incorporated). These results can be explained by the larger particle size of commercial ZnO (0.22 μm), while the nano ZnO (contained in samples ZnO/CAT and ZnO/CA) presented a lower particle diameter (17 nm). Another study [13] has also described the TPP effect, including the decrease of particle size when crosslinker concentrations were increased, which allows to enhance ZnO protection in the simulated gastric fluid.

**Table 2:** Antimicrobial activity of commercial ZnO, ZnO nano, ZnO/CAT and ZnO/CA against *Escherichia coli*

Samples	Sample concentration (μg/mL)	Concentration of incorporated Zn^2+^ (μg/mL)^a^	*E. coli* inhibition (%)
Commercial ZnO	200	198	12.7 ± 0.7
	500	495	100 ± 0.00
ZnO nano	250	195	26.7 ± 2.0
	300	234	45.0 ± 0.6
	350	273	100 ± 0.0
ZnO/CAT	1700	173	6.8 ± 0.3
	2000	204	56.0 ± 0.6
	2250	229.5	100 ± 0.0
ZnO/CA	500	63	4.6 ± 0.9
	750	94.5	44.0 ± 1.1
	1000	126	100 ± 0.0

^a^Average concentration of incorporated Zn^2+^ as determined by atomic absorption spectrophotometry: commercial ZnO, 99%; ZnO nano, 78 mass%; ZnO/CAT, 10.2 mass%; ZnO/CA, 12.6 mass%

**Table 3:** Antimicrobial activity of commercial ZnO, ZnO nano, ZnO/CAT and ZnO/CA against *Staphylococcus aureus*

Samples	Sample concentration (μg/mL)	Concentration of incorporated Zn^2+^ (μg/mL)^a^	*S. aureus* inhibition (%)
Commercial ZnO	200	198	45.6 ± 1.3
	500	495	63.5 ± 1.4
	650	643,5	100 ± 0.00
ZnO nano	150	117	22.7 ± 1.1
	200	156	58.3 ± 1.0
	250	195	100 ± 0.0
ZnO/CAT	1000	102	4.6 ± 0.6
	1250	127.5	16.6 ± 0.9
	2000	204	100 ± 0.0
ZnO/CA	500	63	6.3 ± 0.2
	750	94.5	43.8 ± 0.2
	1000	126	100 ± 0.0

^a^Average concentration of incorporated Zn^2+^ as determined by atomic absorption spectrophotometry: commercial ZnO, 99%, ZnO nano, 78 mass%; ZnO/CAT, 10.2 mass%; ZnO/CA, 12.6 mass%

## Discussion

The addition of TPP (Fig. 1c) to the formulation interfered in the acquired reflections, where the ZnO/CA samples showed higher amounts of amorphous structures, a sodium alginate (SA) characteristic, as observed in Fig. 1b, line I, corroborating data from a previous study [14].

The band shift attributed to the asymmetrical stretching vibrations of the carboxylate group, acquired by FTIR, reported the composite formation, where a linkage occurs between the SA carboxylate groups and the CH amine groups, but only a slight shift (from 1613 cm^−1^ for SA to 1612 cm^−1^ from the ZnO/CA composite) was observed (the band is broader and more intense). Interestingly, a much more expressive shift to lower wavenumbers (from 1613 cm^−1^ and 1419 cm^−1^for SA to 1600 cm^−1^, and 1407 cm^−1^ for ZnO/CAT composite, respectively) of both bands was observed in the ZnO/CAT spectra (Fig. 2b, trace III). This clearly demonstrates the TPP contribution in increasing interactions in this composite.

Moreover, the spectra obtained for these products corroborated the FTIR data, reinforcing the role of TPP in increasing physical interactions between the gel components. The CAT matrix forms a stronger gel than CA (without TPP). On the other hand, the addition of ZnO (Fig. 3b) seems to disturb the gel structure. ZnO/CAT showed G’ and G” values superior to those for ZnO/CA.

The ZnO/CAT complex showed a smooth and fine surface, corroborating a previous report by [15], which may be due to interfacial interactions between the CH chains and nano ZnO, which could possibly act as intermolecular crosslinks. Therefore, ZnO nano can contract and restrict the mobility of CH chains, and subsequently change the expansion of the surface morphology. On the other hand, when observing Fig. 4c, a rougher and crowded surface was observed, probably due to the lack of TPP. This reveals the influence of the addition of TPP on ZnO/CAT morphology (Fig. 4b), in addition to ZnO linking with CH. The addition of TPP decreased the particle size, probably due to crosslinking of polymeric chains, resulting in greater particle compression.

The high performance of the ZnO/CAT sample in SGF can be explained by the addition of TPP, the crosslinking agent, used in the preparation of CH due to its rapid gelling ability [16], where TPP reduces the gel pore size and effectively traps Zn^2+^ in the interstices of the support matrix [16, 17]. In simulated enteric juice, the ZnO/CAT and ZnO/CA complexes released more Zn^2+^ than ZnO nano and commercial samples, as expected. The ZnO delivery systems developed in this study allow for high concentrations of Zn^2+^ in the enteric environment, which are necessary to achieve the antimicrobial effect. The partial dissolution of ZnO particles releases zinc ions (Zn^2+^), which contributes to the antimicrobial activity of the oxide [3] against the enteric microbiota of weanling pigs.

The Zn^2+^ dissociation from the ZnO composites is due to the formation of strong electrostatic but reversible bonds, even without the use of any covalent crosslinking agent [18]. Furthermore, the SA layer could have prevented the gastric degradation of CH, because the positive charges from CH reduced solubility at low pH. However, at around neutral pH, a higher amount of negative charges sequester positive CH charges, resulting in dissolution of the CH/SA complex and increase in the release of Zn^2+^ in the middle enteric region [19]. ZnO/CA and ZnO/CAT showed good release of Zn^2+^ in SEF, triggered by the change in the pH.

The lower concentrations of ZnO/CA are probably due to the absence of TPP, thus allowing closer contact between Zn^2+^ and the target microorganisms, where ZnO/CA showed a complete inhibition of *E. coli* growth at lower concentrations than ZnO/CAT (126 μg/mL Zn^2+^ incorporated against 229.5 μg/mL). For *S. aureus,* ZnO/CA prevented the growth complete at a lower incorporated concentration of Zn^2+^ (126 μg/mL), compared to the 204 μg/mL of ZnO/CAT. The CH/SA complex network, even in the absence of TPP, showed greater efficacy when compared to pure ZnO nano, which is effective in inhibiting *E. coli* and *S. aureus* at concentrations of 273 μg/mL and 195 μg/mL, respectively.

A comprehensive mechanism for the antimicrobial effect of nano-ZnO particles is still under debate. In an aqueous environment, the ZnO NPs aggregate to micrometer-sized particles and do not interact effectively with microorganisms. Thus, the antimicrobial activity has been mainly attributable to the dissolved zinc species [20–24]. The dissolution of ZnO nanoparticles and Zn^2+^ release seem to be able to generate radical oxygen species (ROS), consequently, activating a cytotoxic cascade of events that include intracellular calcium flux, mitochondrial depolarization, and plasma membrane leakage [25–28].

In the conditions of the present study, *S. aureus* was shown to be more sensitive to both the ZnO nano and ZnO/CAT samples in comparison to *E. coli*. The difference in antibacterial activity against both microorganisms can be related to differences in the chemical and structural composition of the cell membranes, particularly of the cell wall [29, 30] and to the preparation of the composites. *S. aureus* tends to develop defenses against oxidative stress by releasing products such as superoxide dismutase, catalase and thioredoxin reductase [31–33]. In our study, we can suggest that the new particles of ZnO were able to assemble at cellular membrane and damage it, despite the greater defense of *S. aureus* to the toxicity of ZnO.

Some ZnO particles can attach to the bacterial membrane surface, damaging it, and this mechanical damage has been postulated as a probable mechanism of antimicrobial inhibition [31]. According to the literature, ZnO nano bacteriostatic effectiveness can be ascribed to the disorganization of the membrane, increasing its permeability and resulting in the internalization of the nanoparticles. One possible explanation is that the antibacterial activity exhibited by ZnO composites is based on the abrasive texture of the ZnO surface. This abrasive texture, due to surface defects, fixes ZnO to the protein molecules from microorganism membranes, promotes internalization and consequent cell metabolism inhibition, killing the bacteria [34].

Several factors can interfere with the daily feed intake by animals, such as diet composition, the quality of raw materials and the sanitary conditions of farms [35, 36]. According to the health challenge of each farm and immune conditions of piglets, different concentrations of ZnO in their diets can be adopted, which usually range between 3000 and 5000 ppm (mg/kg). Considering an average daily intake of 400 g of feed by animal, established according to their growth phase (weaning- 21 days) [37–39], containing 4000 ppm (mg/kg) of the commercial ZnO, it should be expected the release of approximately 31.7 mg of Zn in the piglet gut, an amount 6 fold less than that, which corresponds to the MBC against *E. coli*, 198 mg Zn/0.4 kg of feed, and 8 fold less than MBC for *S. aureus* − 257.4 mg Zn/0.4 kg of feed. Released Zn2+ seems to be under the effective MBC threshold, particularly because considerations about the water intake and gut secretion should be done in order to calculate Zn2+ concentration in the gut.

The ZnO/CAT composites obtained in this study, at the same concentration of ZnO, led to a release of 7.4 mg of Zn, a value 12-fold lower than the MBC for *E. coli* (91.8 mg Zn^2+^) and 11-fold lower than for *S. aureus* (81.6 mg). However, ZnO/CA, released 8.6 mg Zn^2+^, which is 5.8-fold lower for the MBC for both evaluated bacteria (50.4 mg). Besides, the coating of ZnO showed a gut empowering effect due to the reduction in the release of Zn^2+^ in simulated porcine gastric fluid. This feature can reduce the amount of ZnO composites to be administered to weaned piglets.

These experiments revealed a remarkable advantage of the synthetized products compared to commercial ZnO, since the concentration of Zn in pig intestine released from those compounds should be higher. The intake dosage of 9.9 g of commercial Zn should be enough to ensure the release of 198 mg in the middle intestine, corresponding to the MBC value for *E. coli*. In the same way, the intake of 12.9 g of Zn might release 257.4 mg corresponding to ZnO MBC for *S. aureus*. These values ​​are approximately 5 times higher than necessary (2 g) of ZnO/CAT to obtain a release of 91.2 mg that are enough to inhibit *E. coli* and 7 times greater than the intake (1.8 g) necessary to release 81. 6 mg Zn from ZnO/CAT and inhibit *S. aureus* in the gastrointestinal environment. The same would be true for ZnO/CA in a more pronounced way, because in order to release 50.5 mg of Zn (MIC of ZnO/CA to both bacteria) it would be necessary for each piglet to ingest at least 1.2 g of Zn, value 8 and 11 times lower than that observed for commercial ZnO inhibit *E. coli* and *S. aureus*, respectively. Although, the intake of ZnO composites can alter the gut microbiota composition, reducing the bacterial diversity and effecting even the probiotic bacteria, the overall supplementation effect of wearing piglets is positive since it can prevent attachment of pathogenic bacteria to the intestinal villi avoiding wearing unspecific diarrhea.

## Conclusion

We have successfully synthesized new ZnO nanoparticles - ZnO/CA and ZnO/CAT, using CH/SA and an ultrasound-assisted methodology. The novel composites showed an optimum in vitro release profile of Zn^2+^ in simulated enteric fluids assays and expressive antimicrobial activities against *E. coli* and *S. aureus*. The physico-chemical analysis and in vitro assays indicate that ZnO microparticles are a promising designed product to be applied for piglet livestock. The data from this study suggest that the ZnO nanoparticle composites may be effective in diets fed to weanling pigs, and may have the potential to significantly reduce fecal Zn excretion. Experiments with weanling pigs fed diets containing ZnO nanoparticle composites should be conducted to address the clinical evaluation of ZnO composites in piglets fed in controlled conditions.

## Abbreviations

CH: Chitosan
MBC: Minimal bactericide concentration
SA: Alginate
SA, ZnO/CA: ZnO plus both CH
SEM: Scanning electron microscopy
SES: Simulated enteric juice solution
SGS: Simulated gastric juice solution
TGA: Thermogravimetric analysis
TPP: Sodium tripolyphosphate
TPP, ZnO/CAT: ZnO plus CH, SA
ZnO: Zinc oxide
